# Cueing healthier alternatives for take-away: a field experiment on the effects of (disclosing) three nudges on food choices

**DOI:** 10.1186/s12889-019-7323-y

**Published:** 2019-07-22

**Authors:** Tracy T. L. Cheung, Marleen Gillebaart, Floor M. Kroese, David Marchiori, Bob M. Fennis, Denise T. D. De Ridder

**Affiliations:** 1 Department of Social, Health and Organisational Psychology, Utrecht University, PO Box 80140, 3508TC Utrecht, The Netherlands; 2 Department of Marketing, University of Groningen, Nettelbosje 2, 9747AE Groningen, The Netherlands

**Keywords:** Nudging, Food choice, Salience, Social proof, Transparency

## Abstract

**Background:**

The current field experiment demonstrates the effectiveness of *nudging* to promote healthy food choices.

**Methods:**

Three types of nudges were implemented at a take-away food vendor: 1) an accessibility nudge that placed fruits at the front counter; 2) a salience nudge that presented healthy bread rolls to be more visually attractive; and 3) a social proof nudge that conveyed yoghurt as a popular choice. We additionally assessed whether nudging effects would remain robust when a disclosure message was included. The field experiment was conducted over a seven-week period. The measured outcome was the sales of the targeted healthy food products.

**Results:**

The accessibility nudge significantly increased the sales of the fresh fruits. The impact of the salience nudge was limited presumably due to existing preferences or habits that typically facilitate bread purchases. As the sales of the yoghurt shakes remained consistently low over the seven-week period the impact of the social proof nudge remained unexamined. Critically, disclosing the purpose of the nudges did not interfere with effects.

**Conclusions:**

Current findings suggest nudging as an effective strategy for healthy food promotion, and offer implications for topical debate regarding the ethics of nudges.

## Background

There is an urgent need to counter unhealthy eating on a societal level as the growing prevalence of overweight and obesity contributes majorly to the rise of non-communicable diseases (e.g., cardiovascular diseases, diabetes) that not only pose increasing financial strain on healthcare systems [1], but even more worryingly lead as a cause of death worldwide [2]. Many common public interventions aiming to encourage healthy diets or decrease unhealthy eating behaviours have the objective to provide information (e.g., education on what constitutes a healthy diet; caloric and nutrition labelling) to consumers so that they can make more informed, and hence healthier food choices. However, information-based interventions have been largely unsuccessful in achieving actual and sustained behavioural change [3, 4]. Some have attributed the shortcoming of such interventions to their predominant focus on attempting to engage consumers in deliberate and rational thinking, which is at odds with how the majority of food decisions naturally occur [4].

Research has consistently shown that consumers make food choices in a mindless manner with minimal deliberation, with many consumption behaviours occurring outside of awareness often as the result of environmental influences [5–7]. In response, in the current research we employ *nudging*, defined as “any aspect of the choice architecture that alters people’s behaviour in a predictable way without forbidding any options or significantly changing their economic incentives” [8], as an alternative strategy to promote healthier food choices. An example of nudging is strategically placing fruits at the cashier checkout where consumers tend to make impulse purchases to promote sales of healthy snacks. Unlike information-based interventions, nudging bypasses the need for consumers to engage in deliberate and effortful processing, and instead relies on subtle changes to the choice setting to facilitate the ease and convenience with making a healthy choice, so that even a mindless choice could be a healthy one. This inherent characteristic of nudging is a competitive advantage that makes it a more compatible and effective strategy than information-based interventions to promote healthy consumption behaviours. Accordingly, the primary objective of the current research is to examine the effectiveness of three nudging strategies (i.e., presenting healthy food products to be more accessible, more visually salient, and perceived as more popular) at a take-away food vendor in promoting consumers’ purchases of healthy food products. Moreover, we assess whether the effectiveness of nudges hinge on consumers being unaware of their intended purpose. To this end, we test whether using a simple message to disclose the purpose of a nudge might affect its impact.

Nudging interventions are based on the theoretical rationale derived from dual-process models of behaviour. Contemporary dual-processing models posit that behaviours result from the interaction of two modes of processing: an unconscious, fast, and automatic mode (System I) on one hand, and a slow, conscious, and deliberative mode (System II) on the other hand [9, 10]. System I processing occurs by default and effortlessly through associations, heuristics and intuition, and should the need arise then such automatic reactions could be halted or modified by the more effortful and deliberate processing of System II that is guided by goals, explicit beliefs and intentions [10]. While System I processing suffices for getting by day-to-day or routine situations, it is nonetheless prone to cognitive biases and errors in judgements as it heavily relies on environmental cues [10]. As such, System I processes are commonly also described as ‘impulsive’ in some dual processing models [11, 12] and tend to result in suboptimal behavioural outcomes and decisions.

In accordance with this theory, while many consumers intend to eat healthily and express weight concerns [13], much of their food decisions and eating behaviours are driven by habit, affect, impulse, or even spontaneous reactions to the environment as opposed to conscious and careful deliberation [5, 14]. Indeed, the accumulating scientific evidence more generally indicates that, despite having good intentions the majority of behaviours frequently occur on a non-conscious, automatic basis [6, 15, 16]. Research has shown that, for example, feeling hungry [17], being mentally distracted [18] or having engaged in effortful exertions of self-control (i.e., ego-depletion; [19]) could all undermine System II processing. Nudging alludes to the increasing recognition that interventions for behaviour change should target the automatic, quick, and non-conscious mechanisms rather than rely on information and persuasion [20, 21].

### Examples of nudging in public eating environments

The use of nudging interventions has increasingly attracted interest from several governments [22, 23], nonetheless systematic reviews suggest that more evidence of nudging interventions specifically in healthy eating promotion in public spaces is still needed before drawing confident conclusions about their effectiveness [24]. In response, the current field experiment aims to add to this body of research, in which we employ three nudges to promote healthy food choices. The selected nudging strategies (i.e., accessibility, salience, and social proof) can easily be employed in public spaces to promote healthy eating and have demonstrated initial success in doing so.

#### Accessibility

The accessibility to food on the basis of physical proximity influences people’s consumption of that food, such that people tend to consume a greater amount of food that is closer in proximity compared to food that is further away [25]. The assumption for this behaviour is that greater distance involves more effort for obtainment [26]. Moreover, it has also been proposed that the accessibility of food moderates the activation of eating-related information (i.e., affordances), such that food items within physical reach (vs. distant food) more strongly trigger eating affordances that underlie actual consumption behaviour [27]. Accordingly, repositioning food products to be more (or less) accessible by means of altering proximity can increase the intake of healthy food products or in contrast decrease the consumption of unhealthy products. For example, fresh fruits located next to cash registers were more likely to be purchased [28, 29] and the intake of candies and potato chips at the cafeteria decreased when they were repositioned to be further away from cash points [30]. In the current field experiment study, we employed an accessibility nudge to improve the physical convenience for purchasing fruits (i.e., fresh fruits that were initially out of physical reach of consumers were relocated next to the cashier where consumers have direct access) to encourage consumer to purchase more fruits.

#### Salience

People have a natural tendency to approach objects that they find rewarding [31], and the visual quality of a food item can also heighten the motivation for intake [32]. Intriguingly, research has shown that even the mere sight of food can stimulate unplanned consumption behaviour [33, 34]. Building on these research insights, interventions have relied on enhancing the visibility and attractiveness of healthy food products as a strategy to promote their consumption. For example, enhancing the visual presence of healthy snacks at the cash checkouts by increasing their overall quantity at the top of opened shelves generated more sales of the healthy snacks at a hospital cafeteria [35] and placing healthy beverages at eye level in refrigerators also introduced greater sales of these items at hospital cafeteria [36]. In the current field experiment, we used a salience nudge to enhance the visibility and visual attractiveness of healthy bread rolls (i.e., by placing them in a separate container decorated with a green-chequered cloth and a picture of a wheat field) to nudge consumers into preferring these healthy bread rolls over the unhealthier alternatives.

#### Social proof

The food choices of others often have a strong influence on people’s own consumption decisions, and the operation of social norms has been proposed as a mechanism underlying such influence [37]. The social proof heuristic functions as a mental shortcut in the decision-making process and thereby influences behaviour especially in situations where people are not engaged in full cognitive capacity [38, 39]. The provision of descriptive norms regarding the food choices of others has shown to be a successful strategy in encouraging healthier food consumption. For example, presenting a poster denoting that “Everyday more than 150 students have a tossed salad for lunch here” led to significantly more purchases of salads at a university campus cafeteria [40] and installing placards on grocery shopping carts informing the average number of fresh produce bought and the most common fruits and vegetables sold at the supermarket also resulted in a higher proportion of fresh produce purchased [41]. In the current field experiment, we installed a social proof nudge to convey an explicit descriptive norm (i.e., “Bestselling choice”) suggesting the yoghurt shake as the most popular choice amongst customers to encourage its sales.

### Transparency

The goal of nudging in healthy food promotion is to redirect an automatic and mindless choice towards a healthier outcome by changing the environment in such a way that the healthy choice becomes a more convenient, attractive, or normal choice, all in the interest of consumers [42]. Correspondingly, this view has led to some criticism that nudging is only effective if people are not cognizant of being influenced [43]. The underlying premise is that disclosing the intended purpose of nudges may trigger psychological reactance [44], in which people deliberately resist their influence in reaction to feeling manipulated or having their freedom of choice threatened.

Nonetheless, there is scarce research systematically evaluating whether nudging strategies are indeed only effective in covert conditions where consumers are unaware of being nudged. Put differently, it remains an open question whether effects of nudging would still be observed when their purpose is disclosed, potentially stimulating consumers to be more reflective and cognizant in the situation. To our knowledge there is only one field study assessing the effects of the disclosure of nudging specifically targeted at healthy eating promotion, and even so this study has only examined disclosure applied to one type of nudge (i.e., accessibility nudge) [29]. In the current research we investigate whether an accessibility nudge, a salience nudge and a social proof nudge would still be influential when they are accompanied by a disclosure message revealing their presence and intent. Answering this research question not only increases understanding of the drivers behind nudging effects, but also responds to the topical debate surrounding the ethics of employing a strategy (i.e., nudging) that is assumed to operate outside of people’s conscious awareness [45, 46]. Our research findings shed insight by examining whether the provision of a disclosure could be a viable solution to enhance the transparency of nudging.

### The current research

The first objective of the current research was to conduct a field experiment to test the effectiveness of an accessibility nudge, a salience nudge, and a social proof nudge to encourage more purchases of fresh fruits, healthy bread rolls, and yoghurt shakes respectively at a take-way food vendor. Considering that in previous research similar nudges have successfully promoted the purchases of healthy products in student cafeterias [29, 30, 41], in the current study we hypothesize that all three nudges will increase the sales of the targeted healthy options. As a second objective, the current research addresses an underexplored research question by investigating whether nudging effects are robust when their purpose is disclosed. Together, our research findings first and foremost offer relevant practical implications for the design and application of nudging interventions promoting healthy food choices. Furthermore, our findings are also relevant in exploring the provision of disclosure as a viable solution in alleviating ethical concerns over duplicitousness.

## Methods

### Setting and participants

The study took place during February, March, and April 2016, at a take-away food vendor that sold a variety of hot and cold beverages (e.g., coffee, tea, fruit juices, soft drinks, etc.), small meal items (e.g., salads, bread rolls, sandwiches and baked goods), and snacks (e.g., yoghurt, cookies, fruits, etc.) at a large academic hospital in The Netherlands. Participants consisted of all customers (mainly hospital personnel as the location of the take-away vendor was not accessed much by patients) who made purchases at the take-away food vendor during the seven-week period that the field experiment took place.

### Design

The current experiment employed three different nudges to promote the sales of fruits, healthy bread rolls, and a yoghurt shake. The field experiment was designed over a seven-week course such that: 1) Week 1 was a *baseline week* where no nudges were implemented; 2) Week 2 was an experimental *nudge week* where all three nudges were simultaneously implemented (yet targeting different healthy food products) to promote healthy food choices; 3) Week 3, 4, 5, and 6 were *washout weeks* where all three nudges were simultaneously removed to eliminate carryover effects from the previous *nudge week*; and 4) Week 7 was an experimental *nudge and disclosure week* where all three nudges were re-implemented with an additional disclosure slogan conveying the purpose of the nudge.

All purchases were recorded electronically on a weekly basis. The field experiment has been approved by the faculty’s institutional review board.

### Procedure

In both the two experimental weeks (i.e., *nudge week*, *nudge and disclosure week*) the three different nudges including the accessibility nudge, salience nudge, and social proof nudge (see Nudges below) were set up simultaneously at 7.30 a.m. on Monday morning when the take-away food vendor opened, and removed at 5.00 p.m. on Friday when the vendor closed. During the nudge and disclosure week, an additional sign of the disclosure (see Nudge Disclosure below) informing the purpose of the nudge was displayed adjacent to each nudge. The nudges were not implemented during the baseline or washout weeks. After the study had completed its course, the manager of the take-way food vendor provided the electronically recorded weekly sales data of the seven-week period to the researchers.

### Materials

#### Accessibility nudge

**Fig. 1:**
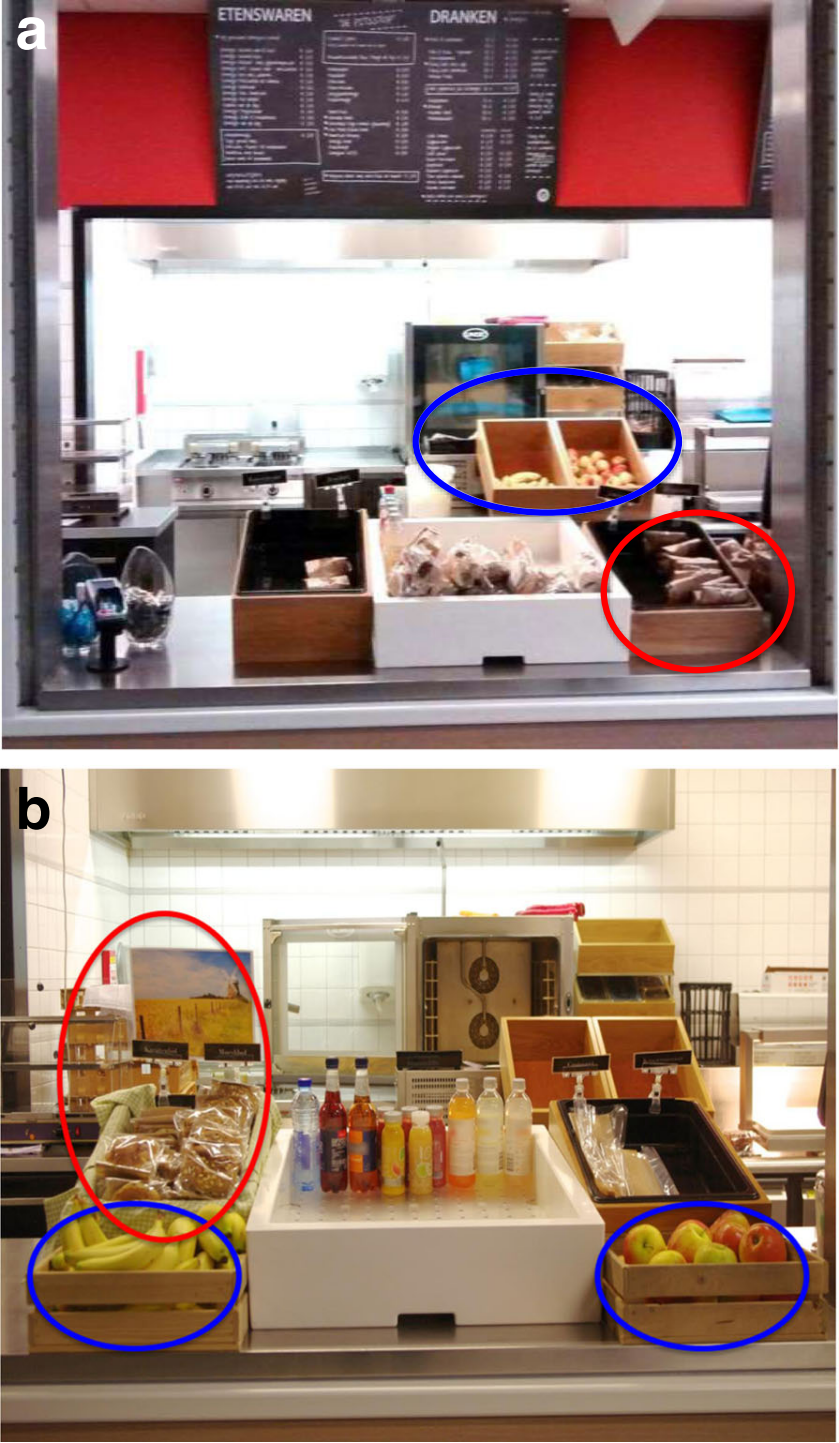
**a** Product arrangement during the baseline week where the fresh fruits were placed in containers at the back of the take-way food vendor and was out of consumers’ physical reach. The healthy and unhealthy bread rolls were placed together in the same container at the front counter. **b** Product arrangement during the nudge week where the accessibility nudge and the salience nudge were installed. The accessibility nudge made the fresh fruits more accessible for consumers by relocating the fruits from the back to the front counter. The salience nudge made the healthy bread rolls more visually salient by placing them in a different container (from the unhealthy bread rolls) decorated with green chequered cloth and a picture of a wheat field

During the baseline week, the fruits were placed behind the counter at the back of the take-away food vendor out of customers’ physical reach (see Fig. 1a). The accessibility nudge removed this physical barrier by placing the fruits at the front counter next to the cashier where customers have direct access (see Fig. 1b). Hence the accessibility nudge aimed to promote the sale of fruit by enhancing the ease and convenience of access for customers.

#### Salience nudge

During the baseline week, the bread rolls with muesli were placed together with the croissants in one container, and the bread rolls with currants were placed in a different container with the cheese croissants (see Fig. 1a). The salience nudge was implemented in order to draw attention to both bread rolls, which were considered the relatively healthier bread options. Hence, the salience nudge rearranged the bread product display by placing both types of bread rolls together in one container, and both types of croissants together in another container. Furthermore, a green-checkered cloth lined the container holding the bread rolls, and a picture of a wheat field was placed on the backside of the container, in order to enhance the overall presentation and salience (see Fig. 1b).

#### Social proof nudge

**Fig. 2:**
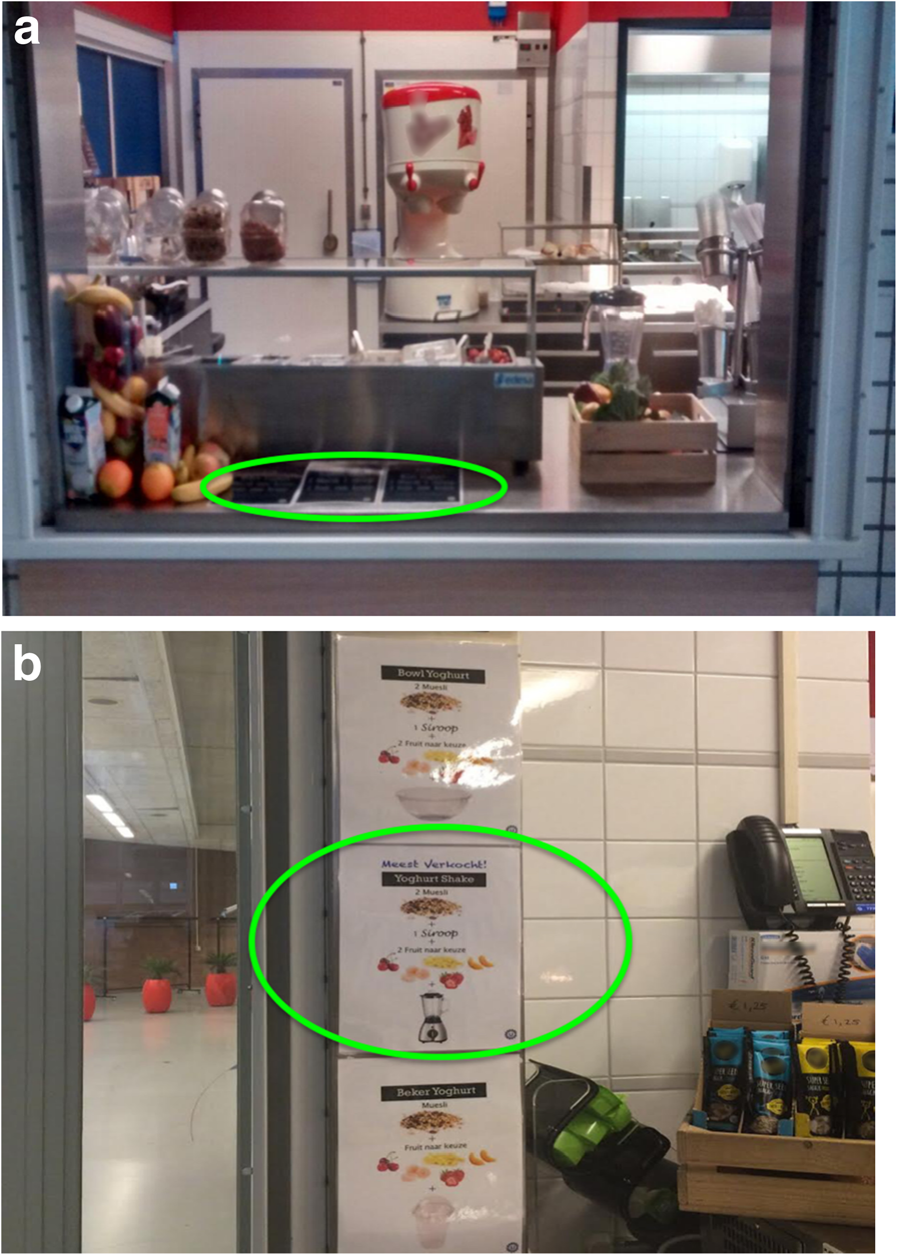
**a** Situation during baseline week where the labels of the yoghurt products were placed flat on the counter. **b** During the nudge week where the social proof nudge was installed, the labels were redesigned to include pictures (e.g., pictures of fruits, muesli, containers) to accompany the text describing the three yoghurt products. Moreover, the labels were placed on the wall at eyelevel. Importantly, the social proof had an additional tagline “Bestselling choice!” to convey a descriptive norm to promote the yoghurt shake

During the baseline week, the labels for the three yoghurt options (i.e., yoghurt bowl, yoghurt cup, and yoghurt shake) were placed flat on the counter. Customers would not have noticed the labels unless they approached the counter (see Fig. 2a). The social proof nudge aimed to promote the yoghurt shake by conveying that it was the preferred choice by the majority of customers. To implement this nudge, the labels for the three yoghurt options was redesigned. First, pictures (e.g., pictures of fruits, muesli, containers) were added to accompany the text to visualize how the three yoghurt options were different from each other. Second, the labels were placed on the wall in clear view. Critically, on the label for the yoghurt shake, an additional tagline “Bestselling choice!” was included to trigger a descriptive norm, thereby providing a social proof heuristic for customers (see Fig. 2b).

#### Nudge disclosure

A small sign with the simple one-sentence message, “We help you make healthy choices”, was displayed accompanying each individual nudge during the *nudge and disclosure week* to disclose the intention of the nudges in place.

### Data processing and analysis

In order to test the effectiveness of the accessibility nudge, the salience nudge, and the social proof nudge respectively, we first present the sales data of the targeted healthy products (i.e., fresh fruits, healthy bread rolls, and yoghurt shakes) collected over the seven-week period (i.e., Week 1: Baseline week, Week 2: Nudge week; Week 3–6: Washout weeks; Week 7: Nudge and disclosure week). We acknowledge that the reported increase or decrease in sales of the targeted healthy products compared between the baseline vs. nudge vs. nudge and disclosure week is only descriptive. Due to the nature of the weekly sales data, which recorded the *total* daily sales of each food product rather than *individual* sales transactions, means and standard deviations could not be calculated, and hence statistical analyses could not be carried out for significance testing to examine the differences in sales between the baseline, nudge, and nudge and disclosure week. Nonetheless, in addition to providing descriptives, we conducted chi-square analyses to test the effectiveness of the respective nudges. Specifically, the chi-square compared the sales of the targeted healthy product to the sales of a comparable unhealthy product between the baseline vs. nudge vs. the nudge and disclosure week.

Additionally, exploratory analyses investigated whether potential spill over nudging effects existed, such that the hypothesized increase of sales for targeted healthy food products would extend from the nudge week to the subsequent washout weeks when the nudges were removed.

## Results

Table 1 presents an overview of the sales of fruits (vs. confectionary), healthy bread rolls (vs. croissants), as well as yoghurt shake (vs. yoghurt bowl and yoghurt cup) across the seven-week course of the entire field study.

**Table 1:** Sales of fresh fruits vs. confectionary; healthy bread rolls vs. croissants; and yoghurt shake vs. yoghurt bowl vs. yoghurt cup; and the total of all sales transactions at the take-away food vendor across the baseline week, the nudge week, the washout weeks, and the nudge and disclosure week

Week	Fresh Fruits	Confectionary	Healthy bread rolls	Croissants	Yoghurt Shake	Yoghurt Bowl	Yoghurt Cup	Total of all sales transactions
Baseline Week	90	142	291	255	7	5	117	14,698
Nudge Week	156	132	318	237	6	7	122	20,921
Washout Week 1	101	129	287	214	3	13	142	12,308
Washout Week 2	140	107	329	209	10	7	136	12,259
Washout Week 3	122	82	310	160	7	9	130	11,579
Washout Week 4	90	82	277	186	4	7	130	13,099
Nudge & Disclosure Week	164	137	327	226	8	9	147	15,579

### The effects of the accessibility nudge on the sales of fruits

During the baseline week a total amount of 90 pieces of fruit were sold. The total amount of fruit sales increased to 156 during the nudge week, which is equivalent to a 73.3% increase. Furthermore, a total amount of 164 pieces of fruit were sold during the nudge and disclosure week. This was a 82.2% increase compared to the baseline week, and a slight increase of 5.1% compared to the nudge week.

Chi-square tests were conducted to test the nudge effectiveness hypothesis. Results are presented in Table 2. We conducted a chi-square test to compare the sales of fruits to the sales of confectionary (e.g., sweets, cookies, energy bars) to examine the impact of the accessibility nudge. Confectionary was chosen as a comparison group because they competed for sales as the ‘unhealthy’ snack alternatives as they were also placed next to the cashier. In line with predictions, the results of the chi-square indicated that there was a significant difference in the proportion of sales between the 3 weeks.

**Table 2:** Chi-square test results for the proportion of fresh fruits vs. confectionary, healthy bread rolls vs. croissants across the baseline week, the nudge week, the washout weeks, and the nudge and disclosure week

	N	χ^2^	*p*
Accessibility nudge (fruits vs. confectionary)			
Three week period	821	16.08	<.001^**^
Three week period + washout	1034	16.73	<.001^**^
Nudge vs. baseline	520	12.18	<.001^**^
Disclosure vs. baseline	533	12.93	<.001^**^
Nudge vs. disclosure	589	.006	.94
Washout vs. baseline	445	9.10	.003^**^
Washout vs. nudge	501	.06	.80
Washout vs. disclosure	514	.10	.75
Salience nudge (healthy bread rolls vs. croissants)			
Three week period	1626	3.67	.16

Note. *P*-values denoted with ^**^ are significant at the alpha = .005 level

Specific pairwise comparisons to examine the sales data of fruits vs. confectionary between the baseline vs. nudge week, baseline vs. nudge and disclosure week, and also nudge vs. nudge and disclosure week. Results from the follow-up pairwise comparisons revealed that the proportion of fruit sales to confectionary sales in the nudge week (fresh fruit: 156; confectionary: 132) was significantly different to the proportion in the baseline week (fresh fruit: 90; confectionary: 142). The proportion of fruit sales to confectionary sales in the nudge and disclosure week (fresh fruit: 164; confectionary: 137) was also significantly different to the proportion in the baseline week. Finally, the proportion of fruit sales to confectionary sales in the nudge week was not significantly different to the proportion in the nudge and disclosure week. Complementing the descriptives, the results from the chi-square analyses demonstrate that the accessibility nudge was effective in promoting fresh fruits (relative to unhealthy confectionaries).

### The effects of the salience nudge on the sales of healthy bread rolls

During the baseline week a total of 291 healthy bread rolls were sold. The total amount of healthy bread rolls increased to 318 during the nudge week, which is equivalent to a 9.3% increase. During the nudge and disclosure week a total of 327 healthy bread rolls were sold, which was a 12.4% increase compared to the baseline week, and a 2.8% increase relative to the nudge week.

We conducted a chi-square test to compare the sales of healthy bread rolls to the sales of croissants to examine the impact of the salience nudge. The croissants were selected as a comparison group because they were the competitive ‘unhealthy’ alternatives in the same product category. Results from the chi-square indicated that the overall differences in proportion of sales in healthy bread rolls compared to croissants was not significantly different between the baseline week (healthy bread rolls: 291; croissants: 245), the nudge week (healthy bread rolls: 318; croissants: 225), and nudge and disclosure week (healthy bread rolls: 327; croissants: 220). While descriptives suggest the sales of healthy bread rolls were higher in the weeks where the salience nudge was implemented, we did not obtain evidence from the chi-square analysis that the proportion of healthy bread rolls compared to croissants was statistically significant different across the three different weeks.

### The effects of the social norm nudge on the sales of yoghurt shakes

During the baseline week, a total of 7 yoghurt shakes were sold. Comparatively, during the nudge week a total of 6 yoghurt shakes were sold, and, a total of 8 yoghurt shakes were sold during the nudge and disclosure week. Considering the descriptives, it was apparent that the sales of the yoghurt shake across the entire seven-week period remained consistently low and would not have warranted sufficient statistical power for analysis. For this reason, statistical analyses were not conducted to examine the effect of the social proof nudge.

### Exploratory analyses of potential spillover nudging effects

In light of the finding that the accessibility nudge significantly increased the sales of fresh fruit in the nudge week compared the baseline week, we explored whether this increase in sales ‘spilled over’ or was sustained in the washout weeks subsequent to the nudge week. Similar to the chi-square analysis used previously, we compared the sales of fresh fruits to the sales of confectionaries between the baseline week vs. nudge week vs. washout week (averaged between the 4 weeks) vs. nudge and disclosure week. The results of the chi-square indicated that there was a significant difference in the proportion of sales between the weeks. Specific follow-up comparisons revealed that the proportion of fresh fruit sales to confectionary sales during the washout week (fresh fruit: 113; confectionary: 100) was significantly different to the proportion in the baseline week (fresh fruit: 90; confectionary: 142). On the other hand, the proportion of fruit sales to confectionary sales during the washout week (fresh fruit: 113; confectionary: 100) was not significantly different to the proportion in the nudge week (fresh fruit: 156; confectionary: 132); nor to the nudge and disclosure week (fresh fruit: 164; confectionary: 137). These results demonstrate that the increase of sales of fruits on the nudge week from the baseline week sustained during the washout-weeks after the accessibility nudge has been removed, thereby suggesting a potential spillover effect of the accessibility nudge.

## Discussion

The automatic basis on which many food choices are made without much deliberation offers a window of opportunity for using choice architectures to gently nudge consumers towards healthy food choices. Specifically we employed an accessibility nudge to increase the convenience for picking healthier fresh fruits, a salience nudge to enhance the visibility and attractiveness of healthy bread rolls, as well as a social proof nudge to promote the popularity of yoghurt shakes at a take-away food vendor located at a university hospital. Considering the sales data, it was evident that the accessibility nudge was a particularly effective nudge in this study. Consistent with previous research findings [25, 28], simply repositioning the fruits from the back to the storefront improved the convenience for picking a fruit and as a result led to a significantly higher proportion of fresh fruits sold compared to confectionaries. Exploratory analyses examined whether the increase in sales of fresh fruits by the accessibility nudge would still be observable in the subsequent washout weeks when the nudge was removed. We acquired some suggestive evidence that the average sales of fresh fruits in proportion to confectionaries were sustained during the four subsequent washout weeks at a level similar to the nudge week, and the nudge and disclosure week. Such finding suggests that even when the fresh fruits were no longer physically and immediately accessible customers still continued to purchase them in relatively greater quantities than confectionaries that were, in contrast, within physical reach. Although this is an intriguing finding it would require considerable replication and future studies should rule out potential confounding factors that may have prompted the ‘spillover effect’ observed in the current study.

The salience nudge was intended to enhance the visibility and visual attractiveness of the healthy bread rolls. Compared to the baseline week it was apparent that in terms of absolute sales of healthy bread, there was a relatively greater proportion of healthy bread rolls sold relative to the croissants in the nudge week, as well as in the nudge and disclosure week. The observed increase was however not significant in statistical terms across the 3 weeks. Nonetheless, these findings do not necessarily dismiss the effectiveness of a salience nudge in general. It may be the case that the influence of the salience nudge was overpowered by consumers’ existing preferences and habits for bread purchases. In an in-store experiment, an accessibility nudge to improve the convenience for purchasing whole-wheat bread did not influence sales [47]. Similar to our cause, the researchers attributed the lack of effect to the strong habitual or planned nature that drives consumers’ bread purchasing behaviour. That said, it would be interesting for future research to examine nudges’ extent of influence in the presence of existing preferences and habits. Lastly, we should once again acknowledge that the low sales of yoghurt shake across the entire seven-week period did not warrant statistical analyses that would be sufficiently powered to examine the effects of the social proof nudge.

In current research, we tested the effectiveness of three nudges (i.e., accessibility, salience, and social proof) in a real-life setting as opposed to a more controlled environment in the lab. We observed that the accessibility nudge appeared to have worked particularly well in promoting a healthy food product in spite of the distractions that were taking place in the setting. On the other hand, due to practical reasons all three nudges were implemented simultaneously in the current study. Future studies could potentially examine whether presenting multiple nudges together would cause interference between the nudges, or whether they could complement each other and have additive effects.

As a second objective, the current study also examined the impact of disclosure. Our findings indicated that disclosing the intended purpose of the nudge did not interfere with its effects (e.g., the sales of fresh fruits in the nudge and disclosure week was comparable to the nudge week), which corroborates recent work [29]. We also did not observe reactance effects – disclosing that the nudge was meant to help consumers make healthy choices did not result in compensation effects or a decrease in the purchasing of unhealthy products. However, in the current study we only disclosed the *intended purpose* of the nudge, and not the actual *presence* of the nudge (e.g., rearranged product placement) in the disclosure message. While our findings suggest that nudging effects remain robust when consumers are made aware of the nudge’s intended purpose through a simple disclosure message, future research should further scrutinize whether this effect still holds when consumers are made aware specifically of the nudge’s presence (i.e., the fact that products were repositioned). Nonetheless, our current finding may be relevant in consideration of the topical debates surrounding the ethics of implementing interventions (i.e., nudging) that may be influencing individuals at large without their awareness. For example, the 2011 House of Lords *Behaviour Change* report [45] published in the United Kingdom asserts that a main criterion for evaluating whether an intervention is ethically acceptable depends on the extent to which it is covert. The report considered two different means to enhance the transparency of the interventions – either through direct disclosure of the intervention or by ensuring that any perceptive person would be able to discern that an intervention (i.e., nudge) has been implemented. The report concluded the latter to be ethically acceptable under the assumption that full transparency might potentially limit the effectiveness of the intervention. However, our research finding actually suggests that it would be viable to disclose the purpose of the nudge with full transparency without undermining its effects. Nonetheless, in the current research we did not assess whether consumers actually read the disclosure message, and therefore would recommend future research to more stringently investigate whether nudging effects would be immune against transparency.

The current study has a number of strengths as well as limitation. A first strength of the study is that the different nudges were tested in the field, therefore allowing for observations in a real-life food choice setting. A second strength pertains to the inclusion of disclosure in this study, as transparency of nudging is heavily debated, but empirical evidence is scarce. Of course, a field study also has limitations. For example, although allowing for real-life food choices, the study did not allow for a controlled setting. Therefore, we are not able to control for other contextual influences on food choice during the experiment. A final note of caution relates to the notion that food *choice* does not automatically mean food *consumption,* which needs to be taken into account when considering implications from this study.

## Conclusion

Our current research has demonstrated nudging to be a low-cost and easy-to-implement strategy to promote healthy food choices. It appears that the fast, non-conscious, and automatic processes are not destined towards unhealthy choices, but could rather be gently nudged by the choice architecture into more optimal, healthy outcomes. These nudging effects are perhaps even immune to conditions when consumers are made aware of being guided toward healthier choices. In closing, with the increasing trend of people eating outside their homes, public eating environments have been identified as strategic places for health promotion [24], and nudging presents itself to be a promising strategy to deliver results in these environments akin to the expression “an apple a day keeps the doctor away”.

## Data Availability

The dataset supporting the conclusions of this article is stored in the institutional repository. Access to this dataset is possible upon reasonable request.
